# Preparation of highly specific monoclonal antibodies against SARS‐CoV‐2 nucleocapsid protein and the preliminary development of antigen detection test strips

**DOI:** 10.1002/jmv.27520

**Published:** 2021-12-21

**Authors:** Chengzuo Xie, Haojie Ding, Jianzu Ding, Yangji Xue, Shaohong Lu, Hangjun Lv

**Affiliations:** ^1^ Institute of Parasitic Disease Hangzhou Medical College Hangzhou China; ^2^ The Key Laboratory of Blood Safety Research, Blood Center of Zhejiang Province Hangzhou China

**Keywords:** colloidal gold immunochromatography, Delta (B.1.617.2) variant, recombinant SARS‐CoV‐2 nucleocapsid protein, SARS‐CoV‐2, the monoclonal antibodies

## Abstract

The coronavirus disease 2019 (COVID‐19) is outbreaking all over the world. To help fight this disease, it is necessary to establish an effective and rapid detection method. The nucleocapsid (N) protein of severe acute respiratory syndrome coronavirus 2 (SARS‐CoV‐2) is involved in viral replication, assembly, and immune regulation and plays an important role in the viral life cycle. Moreover, the N protein also could be a diagnostic factor and potential drug target. Therefore, by synthesizing the N gene sequence of SARS‐CoV‐2, constructing the pET‐28a (+)‐N recombinant plasmid, we expressed the N protein in *Escherichia coli* and obtained 15 monoclonal antibody (mAbs) against SARS‐CoV‐2‐N protein by the hybridomas and ascites, then an immunochromatographic test strip method detecting N antigen was established. In this study, we obtained 14 high‐titer and high‐specificity monoclonal antibodies, and the test strips exclusively react with the SARS‐CoV‐2‐N protein and no cross‐reactivity with other coronavirus and also recognize the recombinant N protein of Delta (B.1.617.2) variant. These mAbs can be used for the early and rapid diagnosis of SARS‐CoV‐2 infection through serological antigen.

## 1 INTRODUCTION

The outbreak of pneumonia due to the severe acute respiratory syndrome coronavirus type 2 (SARS‐CoV‐2) has rapidly spread to become a pandemic all over the world.^1^ And according to the World Health Organization (WHO), there are currently four variants of SARS‐CoV‐2 that impact humans' health worldwide.^2^

From a structural perspective, the SARS‐CoV‐2 has four structural proteins, including nuclear protein (N) that binds genomic RNA to form nucleocapsid, a small envelope protein (E), matrix protein (M), and spike surface glycoprotein (S).^3^ The nucleocapsid (N) protein is the most abundant protein of SARS‐CoV‐2, and it is also one of the pathogenic factors of coronavirus disease 2019 (COVID‐19).^4^ Moreover, the nucleocapsid (N) protein plays a key role at different steps in the replication cycle and is used as a serological marker of infection.^5^ Liu^6^ et al. used N protein as an antigen to detect SARS‐CoV‐2 immunoglobulin M (IgM) and IgG antibodies by enzyme‐linked immunosorbent assay (ELISA), and the results were 68.2% and 70.1% positive, respectively, demonstrating that N protein detection of SARS‐CoV‐2 antibodies is a highly sensitive screening method for the diagnosis of COVID‐19. Serum samples are also generally more stable than viral RNA and less variable than nasopharyngeal or oropharyngeal viral RNA samples due to the uniform distribution of proteins in the blood, minimizing false negatives in test results.^7^ These research studies suggested that the data about the research of nucleocapsid protein may provide a theoretical basis for the diagnosis and treatment of SARS‐CoV‐2 infection.

In this study, the BALB/c mice were immunized with recombinant SARS‐CoV‐2 N protein, anti‐SARS‐CoV‐2 N monoclonal antibodies with high titer and specificity were screened by cell fusion technique. Then, through paired screening of these monoclonal antibodies, a rapid and convenient colloidal gold immunochromatographic strip detection method was prepared, hoping to provide a basis for early diagnosis of clinical novel coronavirus infection.

## 2 MATERIALS AND METHODS

### 2.1 Mice and materials

BALB/c mice were purchased from the Experimental Animal Center in Zhejiang province; mice myeloma cell S/P2.0 were available from our laboratory; Freund's complete adjuvant (FA) and Freund's incomplete adjuvant (FIA) were purchased from Sigma Company; T4 DNA ligase, TMB Chromogen Solution, and Bradford Protein Assay Kit were purchased from Beyotime Biotech; Polyethylene glycol 1500 (PEG 1500) and affinity chromatography (Ni‐NTA Agarose) were purchased from Shanghai Sangon Biotech; the recombinant N protein (Baculovirus‐Insect Cells) and the recombinant N protein of SARS‐CoV‐2 Delta (B.1.617.2) variant were purchased from Sino Biological Inc.

Chlorauric acid is purchased from Shanghai National Pharmaceutical Company; nitrocellulose film (HCN4878711), glass cellulose film (20100701), Polyvinyl chloride (PVC) board (SM31‐25), filter paper (B3MN44840), and packaging shell are all purchased from Shanghai Jinbiao Biotechnology Co., Ltd.; microcomputer automatic cutting machine and XYZ three‐dimensional film‐gold spraying instrument were all purchased from Biodot Company.

### 2.2 Preparation of SARS‐CoV‐2‐N recombinant protein

#### 2.2.1 Plasmid construction

The N gene of SARS‐Cov‐2 (NCBI Reference Sequence:NC_045512.2) was cloned into the prokaryotic expression pUC57 vector by General Biological system Co. The designed primers were: F:5ʹ‐GCCGGATCCATGTCTGATAATGGACCCCAAAA‐3ʹ, R:5ʹ‐GCCGTCGACAGGCCTGAGTTGAGTCAGCAC‐3ʹ. The pUC57‐ SARS‐CoV‐2‐N plasmid was used as a template for polymerase chain reaction (PCR) amplification by the primers. The final PCR products were inserted into the pET‐28a(+) vector digested by restriction enzymes *Bam*HⅠ and *Sal*Ⅰ with T4 DNA ligase at 16°C overnight, then cloned into *Escherichia coli* strain TOP10. The positive pET‐28a (+)‐N plasmid was identified by sequencing and subsequently transformed into *E. coli* strain BL21.

#### 2.2.2 Expression and purification of recombinant N protein

The transformed cells were incubated overnight on Luria Broth agar plate with Kanamycin at 37°C, then picked the single colony to Luria‐Bertani medium containing 50 μg/ml Kanamycin at 37°C. When the OD_600_ reached 0.5–0.8, 1 mM of Isopropyl‐beta‐D‐1‐thiogalactopyranoside (IPTG) was added, and the culture was further incubated for 6 h. Centrifuging the induced bacterial liquid at 8000 rpm/min for 10 min at 4°C, decanting the supernatant, and crushing on ice, the recombinant protein was expressed as inclusion bodies. Decanting the supernatant and resuspending the precipitate with 8 mM urea. Then the bacterial solution was purified by Ni‐NTA column after filtration with 0.45 μm microporous membrane, and the protein was eluted with different concentrations of imidazole. The purified protein was analyzed by 12% sodium dodecyl sulfate‐polyacrylamide gel electrophoresis (SDS‐PAGE) and stained with Coomassie brilliant blue to confirm the pET‐28a (+)‐N expression. And the protein concentrations were determined by Bradford Protein Assay Kit.

#### 2.2.3 Preparation of monoclonal antibodies against SARS‐CoV‐2‐N

Briefly, 20 BALB/c mice aged 4–8 weeks were injected at multiple sites intraperitoneally with 50 μg/mice of diluted recombinant pET‐28a (+)‐N protein then 100 µg/mice for the 2nd and 3rd immunizations every 2 weeks. To check antibody titer, 6 weeks after the first inoculation, venous blood was taken from the tail of mice and the serum titers were determined by indirect ELISA, then the mouse with high serum titers was selected for the subsequent fusion. The BALB/c mice spleen cells were fused with SP2/0 myeloma cells with polyethylene glycol 1500 (PEG 1500) and cultured in HAT‐1640 medium containing Hypoxanthine (H), aminopterin (A), and thymine (T). The indirect ELISA and the limiting dilution‐culture method to screen a stable anti‐SARS‐CoV‐2‐N monoclonal hybridoma cell line. In our experiment, there were 15 stable hybridoma cell lines produced. All mice were maintained under specific pathogen‐free conditions in the Experimental Animal Center in Zhejiang province. The experiments were conducted in accordance with the Guide for the Care and Use of Laboratory Animal.

These 15 hybridoma cell lines were injected into a total of 30 BALB/c mice (2 × 10^7^cells for each mouse) to collect ascites after repeated immunization. Then the ascites of 15 mice were extracted and purified by the caprylic acid‐ammonium sulfate precipitation method. The monoclonal antibodies against SARS‐CoV‐2 N protein were obtained and assessed by SDS‐PAGE.

### 2.3 Characterization of monoclonal antibodies against SARS‐CoV‐2 N protein

#### 2.3.1 Determination of the monoclonal antibody titers

To determine the titer of purified monoclonal antibody (mAbs), recombinant pET‐28a (+)‐N protein was used as coating antigen for 96‐well plates by indirect ELISA method. Then the 15 different mAbs were diluted from 1:10^1^ to 1:10^8^ starting with 1 mg/ml. With the blank hole without mAbs as the negative control, the positive value of OD450 was more than twice that of the negative control, and the highest dilution of mAbs showed a positive result taken as the monoclonal antibody titer.

#### 2.3.2 The paired screening of monoclonal antibodies

In the preparation of antigen detection immunochromatography test strip by double antibody sandwich method, it was necessary to screen a pair of monoclonal antibodies which could specifically bind to SARS‐CoV‐2 N antigen and were not affected by the spatial structure.^8^ Therefore, the recombinant SARS‐CoV‐2‐N protein was diluted as an antigen and labeled with biotin, then the unbound biotin was eluted with a desalting column and the end of the SA sensor was wetted and solidified in the SARS‐CoV‐2 N antigen. Add 200 μl 15 different mAbs, which were diluted with phosphate buffered saline (PBS) and blank controls to the 96‐well plate. The mAbs were detected by the ForteBio Octet Red biomolecule interaction instrument, and the Octet data analysis software CFR Part 11 Version 6.x was used to analyze the data. According to the data, the response value (nm) of each pair of monoclonal antibodies was used as the result of antibody pairing screening. The monoclonal antibody paired screening experiment was carried out by Hangzhou Shuangtian Biology.

#### 2.3.3 Determination of mAbs subtypes

According to the titer of monoclonal antibody and the results of paired screening, the mAbs with high titer and pairing effect were selected to determine the subtypes. And the subtype of the mAbs against SARS‐CoV‐2 N were tested with a Monoclonal Antibody Subtyping Kit (IgG1, IgG2a, IgG2b, and IgG3).

### 2.4 The preparation and evaluation of immunochromatographic test strip

#### 2.4.1 The preparation of colloidal gold solution

To ensure cleanliness, the sterilized 1 L glass flask was rinsed multiple times with deionized water, then added 500 ml deionized water and heated to boil in a magnetic agitator. Dissolved 0.1125 g sodium citrate in 3 ml boiled water in a 15 ml centrifuge tube, subsequently, 5 ml HAuCl_4_ solution (1%) was added with heating and vigorous stirring until the mixture reached a wine red. Then the mixture solution was kept to state for 5 min and stopped, eventually, the solution was naturally cooled to room temperature and store at 4°C.

#### 2.4.2 The preparation of immunogold labeling

According to the results of paired screening of monoclonal antibodies, the antibodies with better results were labeled with a colloidal gold solution.

#### 2.4.3 Labeling colloidal gold solution

The colloidal gold‐mAb conjugation was then performed using optimum pH and antibody quantity. Briefly, mAb with the best labeling quantity was added into 10 ml of colloidal gold solution (adjusted to optimum pH value), and the mixture was stirred vigorously for 30 min in a magnetic agitator. Then, 1 ml Bovine albumin (BSA) (10%) was added to the solution with vigorously stirring for 30 min, and the solution was centrifuged at 12,000 rpm for 30 min, and the colloidal gold solution was resuspended with 500 μl of resuspended solution (containing 20% sucrose, 5% trehalose, 10% BSA). Finally, the colloidal gold‐mAb (SARS‐CoV‐2 N protein) was obtained.

#### 2.4.4 Preparation an assembly of immunochromatographic test strip

##### Preparation of conjugate pad

The labeled colloidal gold‐mAb was sprayed on the fiber membrane by a film‐gold spraying instrument at the amount of 4 μl/cm, which was used as a conjugate pad. Then we placed them into a 37°C oven and were allowed to dry overnight.

##### Labeling the NC membrane

The diluted goat anti‐mouse IgG (1 mg/ml) and the monoclonal antibody (1 mg/ml) were sprayed in the control (C) line and testing (T) line located in nitrocellulose membranes, respectively. Then we also placed them into a 37°C oven and were allowed to dry overnight.

##### The assembly of the immunochromatography test (ICT) strip

According to the structure of the colloidal gold immunochromatography test strip,^9^ the trips were assembled using the labeled colloidal gold‐mAb conjugate pad, NC membrane with test line and control line, PVC base plate, and absorbent pad.

#### The evaluation of the test trips

To detect the sensitivity of the immunochromatographic strips, the recombinant pET‐28a ((+)‐N protein was diluted to 1:2, 1:4, 1:8, 1:16, 1:32, 1:64, 1:128, 1:256, 1:512, 1:1 024, 1:2 048, 1:4 096 with 10 mmol/L PBS solution, and these diluted proteins were simultaneously tested using the immunochromatographic strips to evaluate the sensitivity of them. The lowest concentration that could be detected was the sensitivity of the test strip.

To detect the specificity of the test trips, the recombinant pET‐28a ((+)‐N and the other three coronaviruses were diluted.

To detect if the variant SARS‐CoV‐2 N protein was been recognized by the test trips, the recombinant SARS‐CoV‐2 Nucleocapsid Protein Delta (B.1.617.2) variant was diluted.

## 3 RESULTS

### 3.1 Preparation of SARS‐CoV‐2‐N recombinant protein

**Figure 1:**
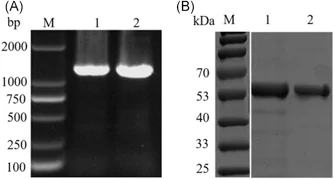
Electrophoresis analysis of PCR products of pET‐28a (+)‐N and SDS‐PAGE analysis of recombinant SARS‐CoV‐2 N protein. (A) M: marker;L1,L2: SARS‐CoV‐2 N gene. (B) M: marker; L1, L2: recombinant SARS‐CoV‐2 N protein. PCR, polymerase chain reaction; SDS‐PAGE, sodium dodecyl sulfate‐polyacrylamide gel electrophoresis; SARS‐CoV‐2, severe acute respiratory syndrome coronavirus 2

The designed primers for the SARS‐CoV‐2 N gene amplified an amost1200 bp band by polymerase chain reaction (PCR), which was observed in agarose gel electrophoresis (1%). (Figure 1A). The recombinant pET‐28a (+)‐N plasmid was transformed into *E. coli* BL21 and the protein expression was induced by IPTG, then the supernatant was purified using Ni‐NTA affinity chromatography after centrifugation. After elution with different concentrations of imidazole, the protein was identified by SDS‐PAGE electrophoresis. As shown in Figure 1B, the band of pET‐28a (+)‐N protein was about 50 kDa.

### 3.2 Preparation and purification of monoclonal antibodies against SARS‐CoV‐2 N protein

**Figure 2:**
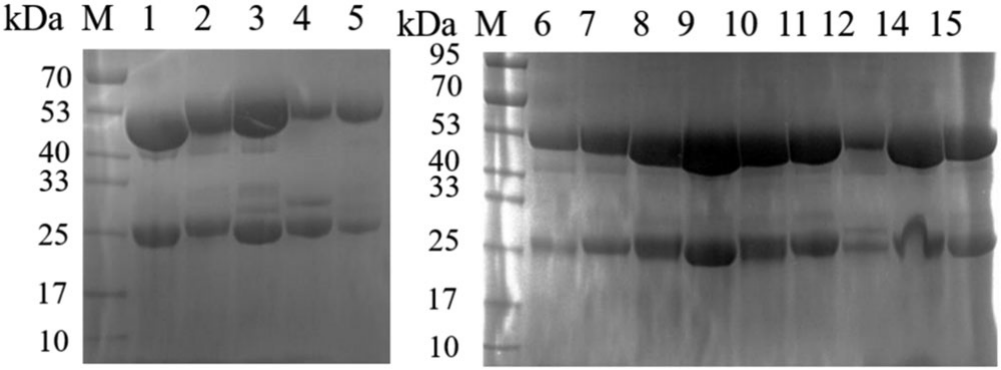
SDS‐PAGE analysis of SARS‐CoV‐2‐N monoclonal antibody. M: marker;L1‐15: the purified mAbs of SARS‐CoV‐2‐N. SDS‐PAGE, sodium dodecyl sulfate‐polyacrylamide gel electrophoresis; SARS‐CoV‐2, severe acute respiratory syndrome coronavirus 2

There were 15 monoclonal antibodies against SARS‐CoV‐2 N protein obtained after immunization, cell fusion, and screening, and they were named N1‐N15. The 15 monoclonal antibodies were purified from ascites by the caprylic acid‐ammonium sulfate method and 14 of them were identified by SDS‐PAGE because of the low concentration of N13 monoclonal antibody. And the N13 monoclonal antibody was removed in subsequent experiments. As shown in Figure 2, the 14 mAbs were composed of heavy chains about 50 kDa and light chains about 25 kDa with obvious bands.

### 3.3 Characterization of the monoclonal antibodies

#### 3.3.1 Determination the titer of monoclonal antibodies

**Figure 3:**
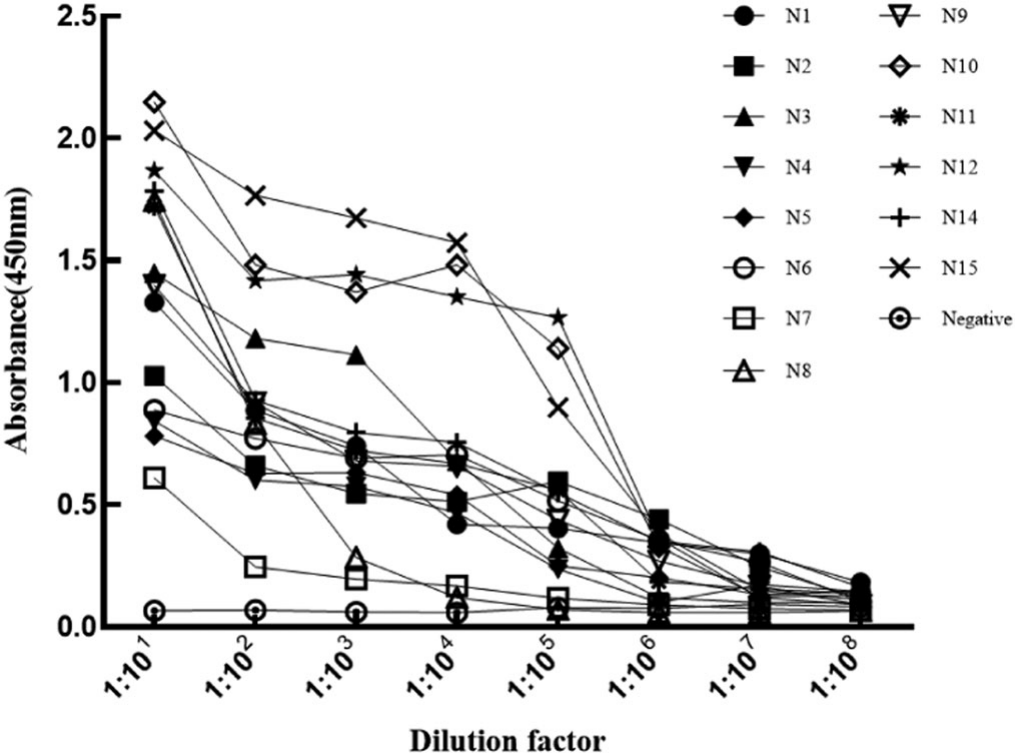
The titer detection of mAbs

The recombinant SARS‐CoV‐2 N protein was coated for a 96‐well plate at 4°C (5 μg/well) overnight, and 14 diluted mAbs were added successively to detect the antibody titer by indirect ELISA method. As shown in Figure 3, according to the value of OD_450_, the N10, N12, N15 monoclonal antibodies with high antibody titer, which were about 1:10^7^, and the titers of other mAbs were about 1:10^6^.

#### 3.3.2 The paired screening of the mAbs

**Figure 4:**
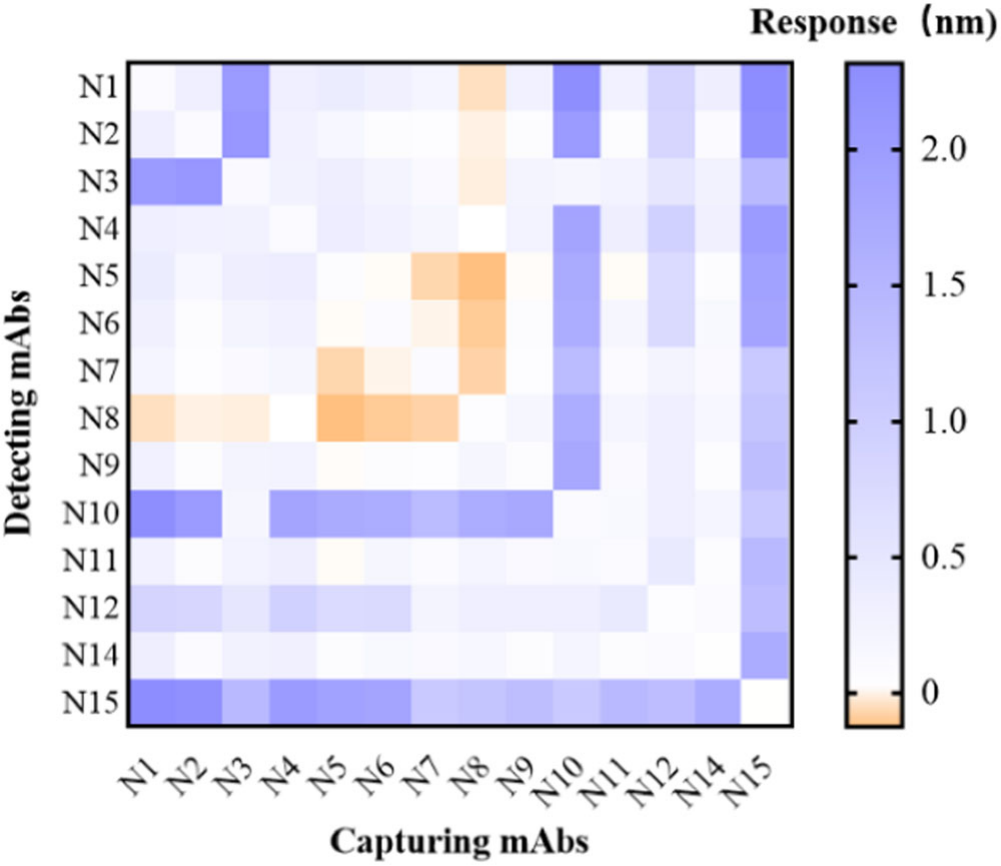
Heat map of paired results of 14 mAbs

The 14 mAbs were determined by paired screening, and the higher the Response value (nm) meant the better the pairing effect. There were the paired screening results of all the 14 mAbs (Figure 4). It could be seen that the N3, N10, N15 were selected for the preparation of the immunochromatographic strips because of their better pairing effect.

#### 3.3.3 The determination of the subtype of the mAbs

**Figure 5:**
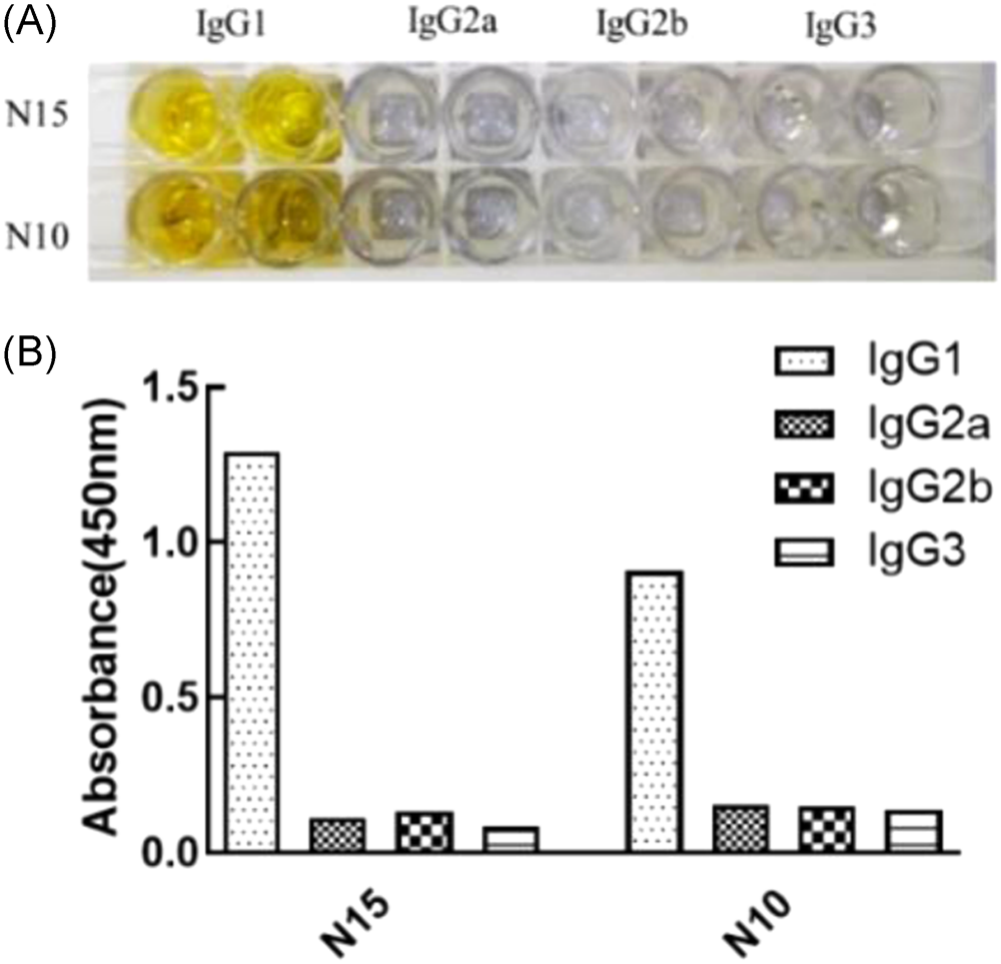
Identification of SARS‐CoV‐2‐N monoclonal antibody subtypes. (A) ELISA identification of antibody subtypes of N15 and N10; (B) the histogram of ELISA identification of antibody subtypes of N15 and N10. ELISA, enzyme‐linked immunosorbent assay; SARS‐CoV‐2, severe acute respiratory syndrome coronavirus 2

The subtypes of N10 and N15 with higher titer and better pairing effect were identified by a Monoclonal Antibody Subtyping Kit. And they were both IgG1 antibody subtyping (Figure 5).

### 3.4 The detection and evaluation of the immunochromatographic strips

#### 3.4.1 The assessment of the sensitivity of the immunochromatographic strips

**Figure 6:**
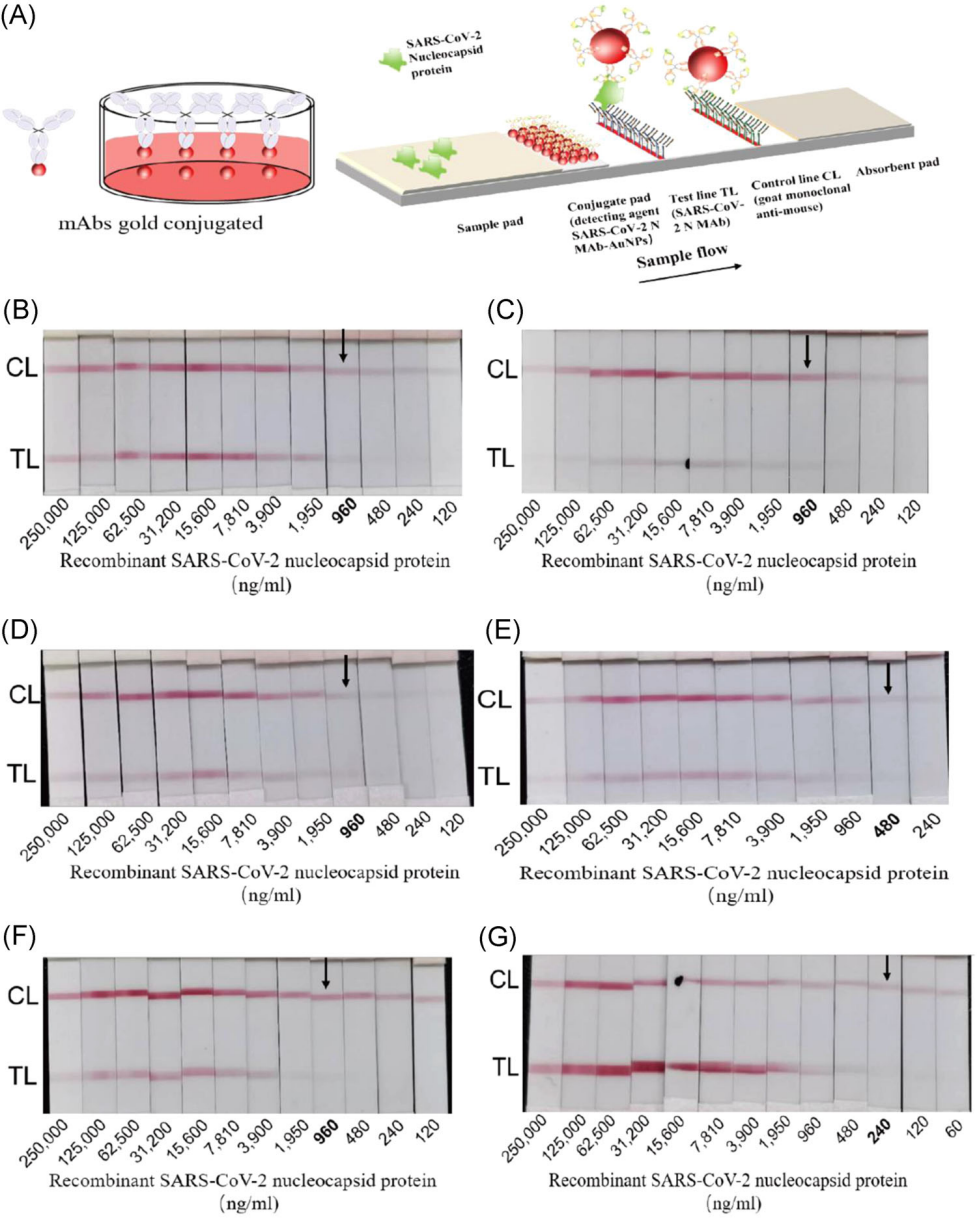
Sensitivity detection of SARS‐CoV‐2‐N antigen dipstick. (A) Schematic representation of the dipstick; (B) N10 gold conjugated, N3 test line, the detection sensitivity is 960 ng/ml; (C) N15 gold conjugated, N3 test line, the detection sensitivity is 960 ng/ml; (D) N3 gold conjugated, N10 test line, the detection sensitivity is 960 ng/ml; (E) N15 gold conjugated, N10 test line, the detection sensitivity is 480 ng/ml; (F) N3 gold conjugated, N15 test line, the detection sensitivity is 960 ng/ml; (G) N10 gold conjugated, N15 test line, the detection sensitivity is 240 ng/ml. SARS‐CoV‐2, severe acute respiratory syndrome coronavirus 2

The antigen detection strips were prepared according to the principal diagram of colloidal gold test strip assembly (shown in Figure 6A), and the test strips were prepared by pairing three monoclonal antibodies N3, N10, and N15 with recombinant SARS‐CoV‐2 N protein antigen at different dilution concentrations were tested, and the sensitivity of different antibody pairs was different. The test strips prepared by colloidal gold labeling with N10 and T‐lines with N15 showed a significant response and the highest detection sensitivity and reached the 12th dilution concentration of 1:2 048, which was weakly positive, the detection concentration was 240 ng/ml. It shows the test strips prepared in this experiment with good sensitivity.

#### 3.4.2 Evaluation of the specificity of the immunochromatographic strips

**Figure 7:**
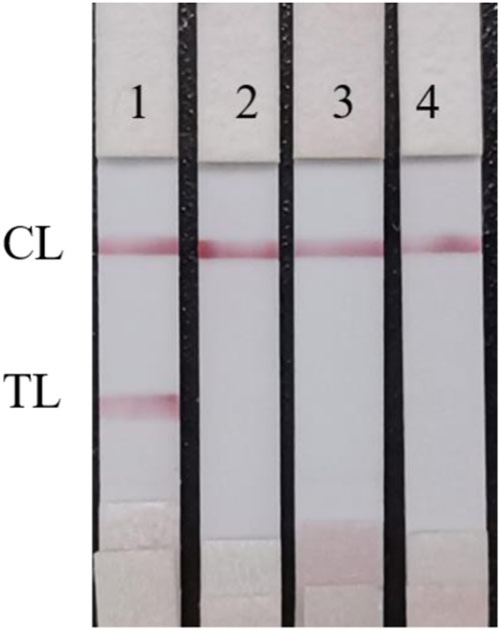
Specificity detection of SARS‐CoV‐2‐N antigen dipstick. 1. Recombinant SARS‐CoV‐2 N protein; 2: Avian infectious bronchitis virus‐HSJ; 3: Avian infectious bronchitis‐SF; 4: Porcine epidemic diarrhea virus. SARS‐CoV‐2, severe acute respiratory syndrome coronavirus 2

As shown in Figure 7, the recombinant N protein showed obvious C and T lines, indicating positive results, while the other three coronavirus detected strips showed obvious C lines only, indicating negative results. It proves the antigen detection test strips prepared in this experiment with good specificities.

#### 3.4.3 The detection of the variants in SARS‐CoV‐2 N protein

**Figure 8:**
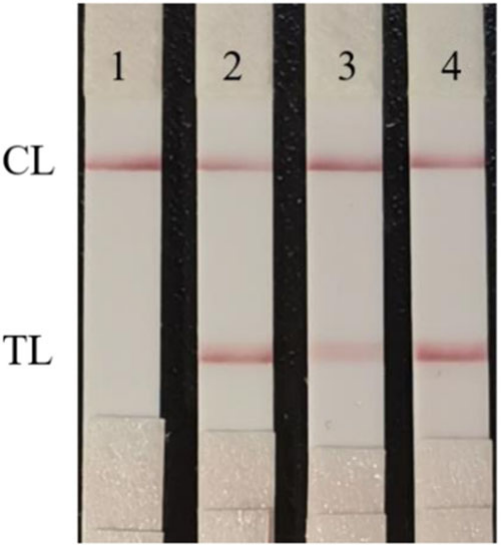
Variant N protein detection of SARS‐CoV‐2‐N antigen dipstick.1. the negative results; 2. the recombinant pET‐28a (+)‐N protein; 3. the recombinant N protein (Baculovirus‐Insect Cells); 4. the recombinant N protein of SARS‐CoV‐2 Delta (B.1.617.2) variant. SARS‐CoV‐2, severe acute respiratory syndrome coronavirus 2

As shown in Figure 8, we detected the recombinant pET‐28a (+)‐N protein (E. *coli*), recombinant N protein (Baculovirus‐Insect Cells), and the recombinant N protein of SARS‐CoV‐2 Delta (B.1.617.2) variant, there were all positive results.

## 4 DISCUSSION

The SARS‐CoV‐2 can cause acute respiratory disease, and the common signs of infection include respiratory symptoms, fever, cough, shortness of breath, and dyspnea. In more severe cases, the infection can lead to pneumonia, severe acute respiratory syndrome, kidney failure, and death.^10^ The SARS‐CoV‐2 mainly spread through the respiratory, so the global epidemic is still spreading and potentially long‐lasting. Early diagnosis and prevention will be a crucial step without specific treatment for novel coronavirus infections.

The current common method diagnostic of SARS‐CoV‐2 is based on the detection of genomic RNA by molecular assays, such as real‐time fluorescent reverse‐transcription polymerase chain reaction (RT‐PCR), which possesses high sensitivity and specificity. However, the sample pretreatments before the experiment were extensive, such as total RNA extraction and high‐cost RT‐PCR reagents, as well as the need for PCR instruments.^11^ These challenges add to some extent to the workload and the risk of infection for healthcare workers. Furthermore, among pathogenic specimens, blood specimens are easier to obtain and more stable than nasal swabs, stool samples, and so on.^12^ According to the recent findings, the SARS‐CoV‐2 N protein is a critical component of the virus replication, involved in the viral particle assembly and the release of viral particles, and a major diagnostic marker of infection and immune protection.^13^ And the antibodies to the N protein are the most sensitive diagnostic marker in the serological diagnosis of SARS infection.^14^ These results suggest that the protein in the serum of SARS‐CoV‐2‐infected patients persistently stimulates the organism for a longer period of time compared with other structures of the virus. Moreover, in the diagnosis of SARS‐COV‐2, rapid antigen testing may present a potential diagnostic advantage because RNA and virion test results maybe not necessarily consistent in the detection.^15^ Therefore, in this study, we expressed and purified SARS‐CoV‐2 N protein, then 15 stable strains of hybridoma cells anti‐N‐protein monoclonal antibodies were obtained, and one mAb with lower concentration was removed in the subsequent experiments, and we detected the titer, paired screening of the remaining 14 mAbs, and developed a SARS‐CoV‐2 antigen test trip based on colloidal gold immunochromatographic strip technique, as shown in Figure 6, the sensitivity could reach 200 ng/ml when detecting recombinant SARS‐CoV‐2 N antigen. Thus, this study can theoretically be used to detect SARS‐CoV‐2 N antigen and to make an early diagnosis of SARS‐CoV‐2 infection.

In this study, several immunological methods such as ELISA based on antibodies to SARS‐CoV‐2 N protein,^16^ immunofluorescence assays,^17^ and test strip methods ^18^ have been established and used for serological testing. However, these antibody‐based assays just reflect SARS‐CoV‐2 infection to some extent, serological antibody diagnosis is mainly based on the detection of IgM or IgG antibodies in the serum. Unfortunately, there is a “window period” for antibody diagnosis and it limits the use of antibodies in the early detection of disease because the period that antibodies emerge after the release of antigen from the virus into the host blood system may take at least 10–28 days.^19^ Li Tao et al. ^20^ quantified SARS‐CoV‐2 antigen and SARS‐COV‐2 IgM/IgG antibodies in serum by ELISA and showed that 76.8% of patients diagnosed with SARS‐CoV‐2 infection were positive for SARS‐COV‐2 N antigen before the appearance of SARS‐COV‐2 N antibodies. Dandan Shan et al. ^21^ used a sensitive Single Molecule Array (Simoa) immunoassay to detect SARS‐CoV‐2 N protein levels in venous blood and showed that the N protein was consistent with the levels detected by the molecule on days 1–7. These results suggested that detection of SARS‐COV‐2 N antigen in serum may be used as an indicator for early diagnosis.

Recently, a novel variant B.1.617.2 has firstly been found in India which increased rapidly in other countries.^22^ And according to a recent study, the Delta variant has an enhanced transmission capacity and increased virulence, in addition, this variant may promote the fusion of the spike protein with cells or inhibit antibodies from binding to it.^23^ In this experiment, the monoclonal antibodies N10 and N15 paired well in the paired preparation of test strips, which could recognize the Delta (B.1.617.2) variant and perhaps the antigen‐binding sites targeted by these two mAbs have potential diagnostic value, then may provide diagnostic targets for the subsequent diagnosis of SARS‐CoV‐2 infection.

## CONFLICT OF INTERESTS

The authors declare that there are no conflict of interests.

## AUTHOR CONTRIBUTIONS

*Formal analysis, methodology, software, investigation, writing–original draft preparation*: Chengzuo Xie. *Conceptualization, methodology, data curation, writing–review & Editing*: Haojie Ding. *Methodology, resources*: Jianzu Ding. *Data curation*: Yangji Xue. *Supervision*: Shaohong Lu. *Funding acquisition, supervision*: Hangjun Lv.

## Data Availability

The genome sequences are available in NCBI (GenBank numbers: NC_045512.2) and other data that support the findings of this study are available from the corresponding author upon reasonable request.
